# Prospects in GSK-3 Signaling: From Cellular Regulation to Disease Therapy

**DOI:** 10.3390/cells11101618

**Published:** 2022-05-12

**Authors:** Sara Arciniegas Ruiz, Ido Rippin, Hagit Eldar-Finkelman

**Affiliations:** Department of Human Molecular Genetics and Biochemistry, Sackler School of Medicine, Tel Aviv University, Tel Aviv 6139001, Israel; smarciniegasr@gmail.com (S.A.R.); idorip.ny@gmail.com (I.R.)

Over the last decade, there has been continuous progress in our understanding of the biology of the protein kinase GSK-3. New data have confirmed the central role of GSK-3 in regulating fundamental biological processes and implicated its involvement in human diseases. In addition, the observation that the hyperactivity of GSK-3 is a potential cause of pathogenesis has caused GSK-3 to become an important drug discovery target in numerous pharmaceutical programs. Indeed, intensive efforts have been invested in developing efficient therapies based on GSK-3 inhibition. Another important challenge addressed in the last decade has been the deciphering of the roles of the two GSK-3 isozymes GSK-3α and GSK-3β. As GSK-3 has been largely overlooked in the past in favor of studies with GSK-3, novel studies now focus on the GSK-3 isozyme, demonstrating different functions from those of GSK-3 in certain scenarios. The contribution of each isozyme to disease pathogenesis is, therefore, a central issue in current studies.

This Special Issue of *Cells* compiles reviews and a research paper presenting updates of the current status and perceptions of GSK-3 in health and disease.

GSK-3 is essential for normal embryonic development, as well as the state of ‘well-being’ in adults, while the aberrant activity of GSK-3 contributes to pathological disease. Rippin and Eldar-Finkelman focus on three pathways representing prominent mechanisms linking GSK-3 with neurodegenerative disorders: the cytoskeleton organization, the mammalian target of the rapamycin (mTOR)/autophagy axis, and mitochondria activity. GSK-3 impairs microtubule (MT) stability and dynamics by phosphorylating microtubule binding proteins, reducing the autophagic flux through its ability to activate mTOR complex 1 (mTORC1), and regulating mitochondria-mediated cell death [1]. Hence, the incorrect regulation of these pathways as a result of GSK-3 hyperactivity impairs normal cellular homeostasis. The inhibition of GSK-3 could, thus, represent a promising therapeutic strategy for treating neurological/neurodegenerative disorders. The authors further suggest that despite the potential discrete functions of GSK-3 isozymes, the inhibition of both isozymes is a favorable approach for the maintenance of the correct balance of GSK-3 activity [1].

Sayas and Avila focus their attention on the contribution of GSK-3 to neurodegeneration via the phosphorylation of the protein tau [2]. The hyperphosphorylation of tau is a well-known hallmark for the formation of neurofibrillary tangles (NFT) in Alzheimer’s disease (AD). The GSK-3 phosphorylation of tau is coupled to the AD -amyloid pathology through a vicious circle, in which -amyloid activates GSK-3 which in turn impairs amyloid precursor protein (APP) processing, and phosphorylates tau [2,3]. Tau hyperphosphorylation, on the other hand, accelerates -amyloid pathology [2] and has deleterious effects on the axonal transport, cholinergic function, and adult neurogenesis, all factors directly related to AD pathogenesis [2]. Therefore, it is not surprising that tau-targeting therapies, including immunization with tau-directed vaccines, immunotherapy with antibodies recognizing various phosphorylated tau epitopes, and GSK-3 inhibitors, are currently at the forefront of AD drug discovery programs [2].

Indeed, the search for adequate GSK-3 inhibitors is still an ongoing challenge. Snitow et al. focus their review on a classic therapy for bipolar disorder, namely, lithium salts (Lithium), identified as a GSK-3 inhibitor some 25 years ago [3] approved for use in the 1970s despite the unfavorable toxic effects that limit its therapeutic window. Pharmacological and genetic manipulations of GSK-3 that reduced GSK-3 activity in animal models phenocopied the effects of lithium activity, supporting the view that GSK-3 is an in vivo target of lithium [3]. Snitow et al. suggest a working model that explains the therapeutic benefits of lithium: the inhibition of GSK-3 by lithium resensitizes cells to respond to extracellular signals. In this model, the negative feedback regulation of GSK-3 on Akt is disrupted by lithium, which enables the activation of Akt (which also serves as a prosurvival factor) through neurotrophins and neurotransmitters that improve mood behavior [3]. The therapeutic benefits of lithium in a variety of animal disease models include improvements in cognitive functions, motor activity, and behavior disorders [4]. As the only approved “GSK-3 inhibitor”, lithium is the subject of clinical trials for a variety of indications, including AD, Huntington’s disease, autism, Fragile X syndrome, Down syndrome, and pain [4].

In addition, Snitow et al. discuss the dual role of GSK-3 in regulating cancer. They suggest that GSK-3 could either promote or inhibit tumorigenesis through the ability to regulate cell apoptosis, cytoskeleton organization, inflammatory signals, and the self-renewal of stem cells, which are all important hallmarks of cancer [3]. Clinical trials with lithium and the GSK-3 inhibitor LY2090314 have not achieved the expected efficacy in treating AML (acute myeloid leukemia) [3]. They also described another timely aspect that concerned the antiviral activity of lithium. Although lithium has been tested against a number of coronaviruses, there is no report of any antiviral activity in treating COVID-19. Some observations support the role of GSK-3 in regulating the viral N protein function, indicating that GSK-3 inhibition may reduce COVID-19 infection capabilities. This, however, needs to be further verified in clinical trials [3].

The engagement of GSK-3 in regulating adult stem cells behavior was reviewed by Racaud-Sultan and Vergnolle [5]. GSK-3 was initially implicated in stem cell self-renewal [6], and shown to play a pivotal role in controlling the self-renewal/differentiation decision [7]. The review describes the function of GSK-3 in the adaptation of adult stem cells to their microenvironment (niche). Under basal conditions, GSK-3 activity is controlled in the niche by adhesion and protease-activated receptors (PARs). The precise balance of GSK-3 activity dictated by the niche impacts the decision of expansion versus differentiation in adult stem cells (e.g., inhibition vs. activation of GSK-3, respectively) [5]. An additional role of GSK-3 in this regard stems from the ability to protect adult stem cells against nutrient starvation and oxidative stress. This capability is of particular importance under pathological conditions, where the niche cannot provide sufficient antiapoptotic protection. Finally, disruptions to the normal niche function, reflected by GSK-3 hyperactivity, may be used as a diagnostic tool to detect aberrant activity of stem cells [5].

Taking into consideration that the evolution of GSK-3 differs dramatically from that of GSK-3, it is important to deepen our understanding of the unique biochemical, structural, and biological properties of GSK-3, the isozyme that has been “historically” overlooked (in favor of GSK-3) [1]. Certainly, some clues can be gleaned from GSK-3 knockout (KO) models. GSK-3 is a key regulator of the glucose metabolism, and its negative regulation of insulin signaling is evidence of an involvement in insulin resistance and type 2 diabetes [8]. Several GSK-3 knockout (KO) models have now been generated in an effort to decipher the roles of the GSK-3 isozymes in regulating glucose homeostasis and obesity. An initial report demonstrated that animals with a genetic embryonic deletion of GSK-3 displayed an increased insulin sensitivity, an increased hepatic glycogen content, and a reduction in body fat mass [9]. The work of Gupte et al. describes a novel line of mice with a globally conditional GSK-3 isozyme-specific deletion [10]. The benefits of this model lie in the ability to obtain live animals with a GSK-3 deletion, since GSK-3 KO is lethal, as well as the possibility to overcome the developmental defects typically caused by the deletion of GSK-3. Their study found that the deletion of GSK-3 isozymes affects the glucose metabolism and body weight. Thus, the absence of GSK-3 improved glucose tolerance (GTT) in insulin-resistant animals fed a high-fat diet (HFD), but did not affect their body weight. In contrast, the deletion of GSK-3 protected the animals from HFD-induced obesity, with a slight improvement in GTT [10]. Notably, the prolonged inhibition of GSK-3 resulted in an increased body weight [10]. Taken together, GSK-3 appears to be the dominant player in causing glucose intolerance under insulin resistance conditions, while GSK-3 controls the fat tissue mass. These effects may be correlated with the phenomenon of body weight gain associated with the lithium treatment. Thus, maintaining a healthy weight is of prime importance for patients on chronic lithium therapy [10].
